# Effects of culture method on response to EGFR therapy in head and neck squamous cell carcinoma cells

**DOI:** 10.1038/s41598-019-48764-3

**Published:** 2019-08-28

**Authors:** Jose M. Ayuso, Ross Vitek, Adam D. Swick, Melissa C. Skala, Kari B. Wisinski, Randall J. Kimple, Paul F. Lambert, David J. Beebe

**Affiliations:** 1 Morgridge Institute for Research, 330N Orchard street, Madison, WI USA; 2 Department of Biomedical Engineering, University of Wisconsin, Madison, WI USA; 3 The University of Wisconsin Carbone Cancer Center, University of Wisconsin, Madison, WI USA; 4 Department of Human Oncology, University of Wisconsin, Madison, WI USA; 5 Department of Pathology & Laboratory Medicine, University of Wisconsin, Madison, WI USA; 6 Department of Oncology, University of Wisconsin School of Medicine and Public Health, Madison, Wisconsin 53706 USA

**Keywords:** Cancer models, Head and neck cancer, Cell death

## Abstract

The EGFR pathway plays a critical role in head and neck squamous cell carcinoma (HNSCC). Targeted therapies against the EGFR are utilized as a treatment for HNSCCC. However, patient response is heterogeneous and molecular biomarkers are lacking to predict patient response. Therefore, functional assays where drug response is directly evaluated in tumor cells are an interesting alternative. Previous studies have shown that experimental conditions modify the drug response observed in functional assays. Thus, in this work the influence of the culture environment on response to Cetuximab (EGFR monoclonal antibody) and AZD8055 (mTOR inhibitor) was evaluated. HNSCC UM-SCC-1 and UM-SCC-47 cells were cultured in 2D monoculture and compared with: 2D co-culture with cancer-associated fibroblasts (CAF); 3D culture in collagen hydrogels; and 3D culture in tumor spheroids. The results showed UM-SCC-1 drug response significantly changed in the different culture environments; leading to an increase in drug resistance in the CAF co-culture and the 3D spheroids. Conversely, UM-SCC-47 exhibited a more constant drug response across culture conditions. In conclusion, this work highlights the importance of culture conditions that modulate response to EGFR pathway inhibition.

## Introduction

Head and neck squamous cell carcinoma (HSCC) is responsible for more than 90% of the malignancies that arise in the mucosal areas in the head and neck^1^. According to the latest statistics, more than 50,000 patients are diagnosed every year with HNSCC; causing more than 10,000 deaths only in the United States (American Cancer Society, www.cancer.org). Epidermal growth factor receptor (EGFR) pathway plays a critical role in HNSCC. In fact, more than 90% of the HNSCC patients exhibit EGFR overexpression^2^. EGFR ligands include EGF, transforming growth factor alpha (TGF-α) and amphiregulin^3,4^. Interestingly, both TFG-α and amphiregulin are also overexpressed in HNSCC; leading to an autocrine activation of the EGFR pathway. Overactivation of EGFR pathway leads to increased cell proliferation, migration, invasion and decreased patient survival^1,3,5^. Thus, the EGFR pathway has been targeted with multiple compounds including EGFR monoclonal antibodies and tyrosine kinase inhibitors^3,5^.

In this context, cetuximab (CTX) (i.e. monoclonal antibody targeting EGFR) was approved in 2006 by the FDA for HNSCC treatment in combination with radiotherapy^6^. Other experimental treatments targeting downstream components of the EGFR pathway have also been tested, including mTOR inhibitors like AZD8055^7^. These targeted therapies against EGFR pathway, combined with chemotherapy/radiation, have become part of the standard approach for many patients with HNSCC^8^. However, patient response is highly heterogenous, in fact only 10–20% of the patients show a favorable response to CTX in monotherapy^9^. Additionally, EGFR expression alone is not a good predictor of patient response to anti-EGFR therapy^2^. Multiple studies have suggested different mechanisms that can explain this variability and the generation of tumor resistance: EGFR polymorphisms and mutations(e.g., EGFR G465R)^10,11^; overexpression of activated receptor downstream tyrosine kinases involved in the EGFR pathway^12^; or downregulation of EGFR pathway inhibitory intermediates (e.g. DUSP5, DUSP6)^13^. Although human papillomavirus (HPV) leads to 25–60% of HNSCC worldwide, it does not have a clear effect on anti-EGFR therapy^14^. Therefore, instead of relying entirely on molecular biomarkers, recent reports are suggesting a more functional approach where drugs are directly evaluated in HNSCC cells^15–17^.

Several reports have shown that *in vitro* drug sensitivity can dramatically differ from *in vivo* observations^17^. In fact, the *in vitro* culture conditions significantly alter drug response compared to *in vivo* conditions^18^. Specifically, CTX sensitivity in colorectal cancer changes when cultured in 2D, 3D hydrogel, and 3D organoid^18,19^. Therefore, to evaluate the possibility of using functional assays in HNSCC, we evaluated the influence of the culture environment on CTX and AZD8055 sensitivity. HNSCC UM-SCC-1 and UM-SCC-47 cells, HPV-negative and HPV-positive respectively, were cultured in different environments: 2D monoculture, 2D co-culture with cancer-associated fibroblasts (CAFs); 3D hydrogels; and 3D spheroids. The cells were exposed to CTX and AZD8055 and cell viability was evaluated; showing the deep impact of the culture environment in CTX and AZD8055 response in HNSCC cell lines.

## Materials and Methods

### Cell culture

UM-SCC-1 and UM-SCC-47 were routinely cultured in DMEM high glucose (Gibco, 11964-092) supplemented with 10% FBS (Thermo Fisher) and Penicillin/Streptomycin (Gibco, 15140). AZD8055 and CTX (Erbitux®) were purchased from LC-Labs and Lilly®. The identity of all cell lines was confirmed within 6 months of use by short tandem repeat testing.

### Patient-derived fibroblast isolation

The research protocol to obtain tumor tissue and isolate fibroblasts following surgery at the University of Wisconsin Hospital (Madison, WI) was approved by the Institutional Review Board. Informed consent was obtained prior to surgery from patients to use residual tissue. All experiments were carried out in accordance with relevant guidelines and regulations. Collagenase Type 1 (Worthington Biochemical) at 5 mg/mL, Dispase (Worthington Biochemical) at 1 mg/mL, and DNase 1 (Worthington Biochemical) at 1000 U/mL were dissolved into Hepatocyte Wash Buffer (ThermoFisher Scientific). This mixture was then sterile filtered using a 0.2 µm syringe filter. Next, head and neck tumor tissue (collected in accordance of the UW-Madison Institutional Review Board) was minced with a handheld razor to 500 µm^3^ pieces. Minced tissue was placed in 1 mL pre-sterilized collagenase digestion buffer in a 5 mL round-bottom polystyrene tube and incubated in a rotating hybridization oven at 37 °C for 4 hours. After digestion was completed, the digestion reaction was neutralized by adding equal volume (1 mL) of hepatocyte wash buffer supplemented with 10% fetal bovine serum (VWR, Radnor, PA USA). Samples were then strained through a 100 uM tube top filter (Corning) and washed with 500 uL 1X PBS. Cell suspensions were centrifuged at 1000RPM for 3 minutes and re-suspended in FM Fibroblast Media (Sciencell). Cultures were maintained at 37 C with 5% CO_2_.

### Co-culture well-plate fabrication and drug response

1500 UM-SCC-1 or UM-SCC-47 cells were seeded in the co-culture well-plate. For co-culture experiments, 1500 CAFs were seeded in adjacent wells and culture media connected both wells; allowing paracrine signaling. After 24 hours, culture media with/without drug was added, and cell viability was measured after 3 days.

### Culture in 3D collagen hydrogels and drug response

In order to embed UM-SCC-1 and UM-SCC-47 cells in the 3D hydrogel, a 4.0 mg/ml collagen hydrogel was prepared as follows: 25 µl of 10X PBS, 5.62 µl of 1 N NaOH; 224 µl of 8.90 mg/ml collagen type I; and 245 µl of cell suspension at 500 cells/µl. 3 µl droplets were placed in a 96 well-plate and collagen was polymerized at room temperature for 20 min. Culture media was added on top and the well-plate was left in the incubator. After 24 hours, culture media with/without the drug was added and cell viability was evaluated after 3 days.

### Spheroid generation and drug response

Tumor spheroids were generated by the hanging drop method described in^20–22^. Briefly, UM-SCC-1 and UM-SCC-47 cells were trypsinized, counted and resuspended at 60 cells/µl in media supplemented with 20% 12 g/l methylcellulose. 25 µl were placed µl droplets were placed on top of a Petri dish lid and distilled water was added to the bottom of the dish to reduce evaporation during the spheroid formation. After 24 hours in the incubator, one single spheroid per droplet was formed. 25 µl of media with/without AZD8055 or CTX were added and the samples were incubated for another 3 days before measuring cell viability.

### Luminescence-based cell viability assay and statistical analysis

Cell viability was evaluated in all the different cell environments using the CellTiter-Glo® 2.0 Assay (Promega, G9241). Briefly, after exposing the cells to CTX or AZD8055 CellTiter-Glo® reagent was added directly to the cells in a 1:1 ratio. This reagent lysed the cells and generated light based on the amount of ATP released by the cells. Samples were incubated for 45 min in the dark and cell-induced luminescence was evaluated in a plate reader. Luminescence intensity linearly correlated with the number of cells in the well-plate. Background intensity (i.e., no cells) was subtracted from the experimental conditions and values were then normalized to DMSO controls. Dose-response curves were plotted with GraphPad Prism v7 using the inhibitor concentration vs response (three parameter) function. A line of fit was created using a non-linear least squares regression. An extra-sum-of-squares F-test was used to analyze the maximum and minimum cell viability as well as curve slope across dose-response curves and calculate p-values.

### Confocal microscopy

AZD8055 and CTX effect on spheroid viability was also evaluated by confocal microscopy. UM-SCC-1 and UM-SCC-47 cells were stained with cell tracker green CMFDA (Thermo Fisher, C2925) prior generating the hanging drops. Briefly, cell tracker green was diluted 1:1000 in the cell suspension and cells were stained for 15 min in the incubator. The cells were centrifuged and resuspended in 15 ml of PBS to wash the excess of cell tracker; this step was repeated twice. Finally, cells were resuspended in growth media with methylcellulose and cultured in the hanging drops. Spheroids were exposed to CTX or AZD8055 and 24 hours before assessing spheroid viability, propidium iodide (PI) (Sigma, P4170) was added to the media to label dead cells in red. Spheroid viability was evaluated using a Leica SP8 STED confocal microscope.

### Image and statistical analysis

Confocal images were analyzed using Fiji (https://fiji.sc/). Drug penetration into the spheroid was calculated by plotting the fluorescence profile across the middle section of the spheroid (yellow rectangle in the images). All the experiments were repeated at least three times independent times. The normal distribution was tested using the Kolmogorov-Smirnov test in GraphPad Prism 7. For parametric comparisons one-way ANOVA test was used.

## Results

### Effect of fibroblasts in CTX and AZD8055 response

**Figure 1:**
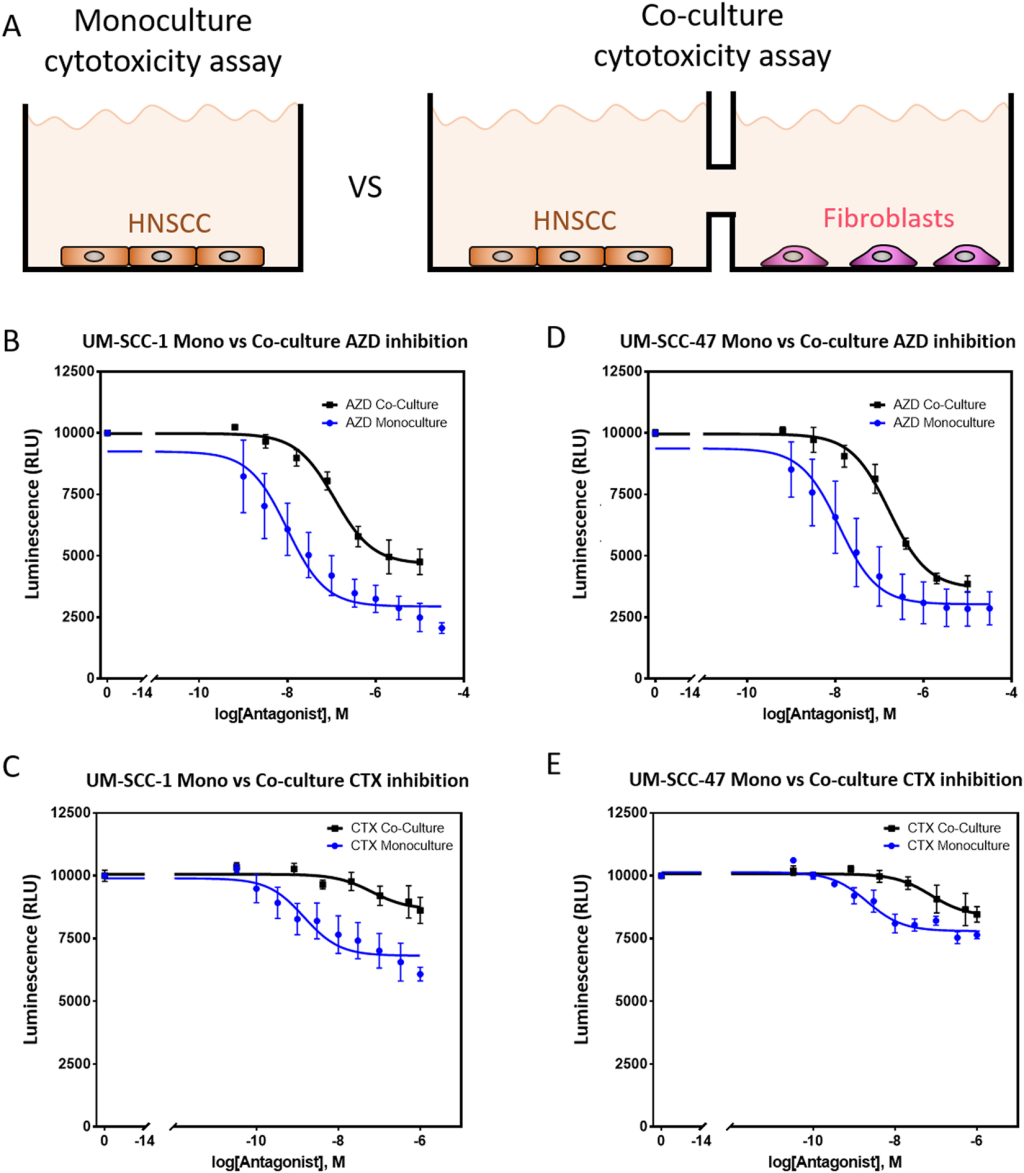
2D mono-culture vs co-culture. (**A**) UM-SCC-1 and UM-SCC-47 were cultured alone or co-cultured with CAFs in a custom 96 well-plate. In the co-culture well-plate, tumor cells and fibroblasts were seeded in adjacent wells that were connected by a liquid bridge; allowing paracrine signaling between the different cell types. AZD8055 or CTX effect was evaluated after 3 days in mono and co-culture conditions. (**B**) Graph shows UM-SCC-1 response to AZD8055 in mono- and co-culture. (**C**) UM-SCC-1 response to CTX in mono- and co-culture. (**D**) UM-SCC-47 response to AZD8055 in mono- and co-culture. (**E**) Graph shows UM-SCC-47 response to AZD8055 in mono- and co-culture. Graphs show mean value ± standard deviation.

The effect of CAFs in drug response was evaluated using a custom co-culture 96 well-plate. This plate allowed the formation of a liquid bridge between adjacent wells; connecting the two cell populations cultured in each well (Fig. 1A). Thus, CAFs and UM-SCC-1 (HPV-negative) or UM-SCC-47 (HPV-positive) cells were seeded, and after 24 hours AZD8055 or CTX were added to the media. The results showed the presence of CAFs significantly changed UM-SCC-1 cell sensitivity to AZD8055. CAFs reduced the cytotoxicity of AZD8055 by nearly 3 orders of magnitude at the highest drug concentration and the cell viability was significantly higher across the dose-response curve slope in the presence of CAFs (p-value < 0.001) (Fig. 1B). Similarly, the presence of CAFs also significantly decreased the cytotoxicity of CTX across the drug concentration range (p-value < 0.001) (Fig. 1C). Next, we evaluated the influence of CAFs in UM-SCC-47 cells. The results showed that the presence of CAFs significantly modified the dose-response curve slope showing increased UM-SCC-47 cell resistance to both AZD and CTX across the drug concentration range (p-value = 0.01) (Fig. 1D,E).

### Influence of 2D vs 3D environment

**Figure 2:**
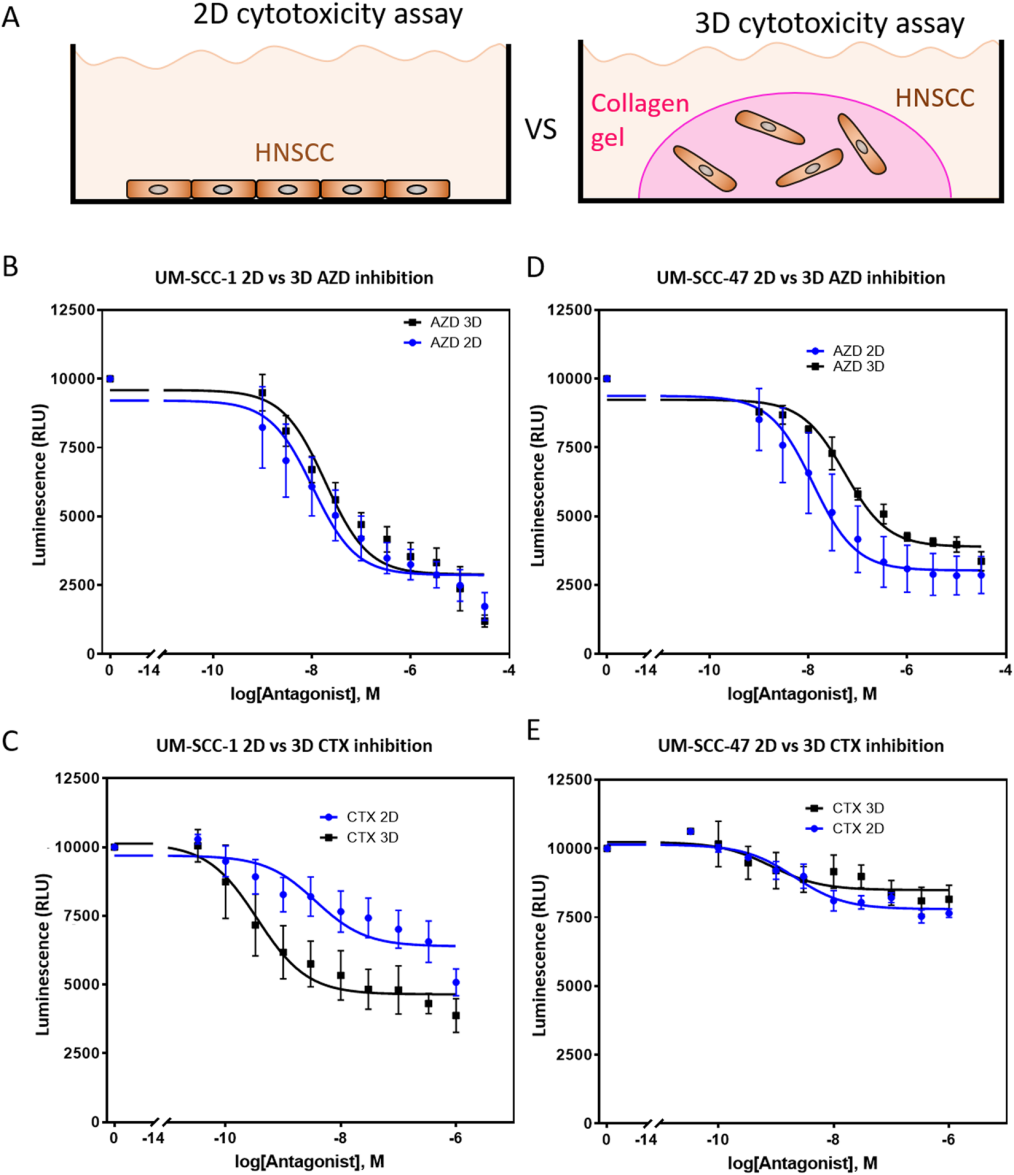
2D vs 3D. (**A**) UM-SCC-1 or UM-SCC-47 were cultured in 2D on a plastic 96 well-plate or embedded in a 3D collagen hydrogel. The cell number and media volume were kept constant between the 2D and 3D experiments. Response to AZD8055 and CTX was evaluated after 3 days. (**B**) Graph shows UM-SCC-1 response to AZD8055 in 2D and 3D conditions. (**C**) UM-SCC-47 response to AZD8055 in 2D and 3D. (**D**) UM-SCC-1 response to CTX in 2D and 3D. (**E**) Graph shows UM-SCC-47 response to AZD8055 in 2D and 3D. Graphs show mean value ± standard deviation.

Next, we set out to evaluate the influence of the 2D vs 3D environment in drug response. UM-SCC-1 and UM-SCC-47 cells were embedded in a collagen hydrogel and cultured for 3 days in the presence of AZD8055 or CTX. Then, cell viability was analyzed and compared with cells cultured in 2D (Fig. 2A). The results showed that UM-SCC-1 cell sensitivity to AZD8055 in 2D and 3D was similar, exhibiting no statistical difference in the cell viability at the highest drug concentration or in the curve profile (p-value = 0.38) (Fig. 2B). Interestingly, UM-SCC-1 cells were more sensitive to CTX in 3D compared with 2D conditions. CTX displayed 1.4 times greater cytotoxicity in 3D than in 2D at highest drug concentration and cell viability was significantly lower across the dose-response curve slope, p-value < 0.0001 (Fig. 2C). On the other hand, UM-SCC-47 cells showed a different pattern. AZD8055 response in 3D was different compared with 2D, showing less cytotoxicity and a significantly different dose-response slope in 3D (p-value < 0.001) (Fig. 2D). Finally, UM-SCC-47 cells showed no change in CTX sensitivity in 3D compared with 2D (p-value = 0.12) (Fig. 2E).

### Spheroid drug response

**Figure 3:**
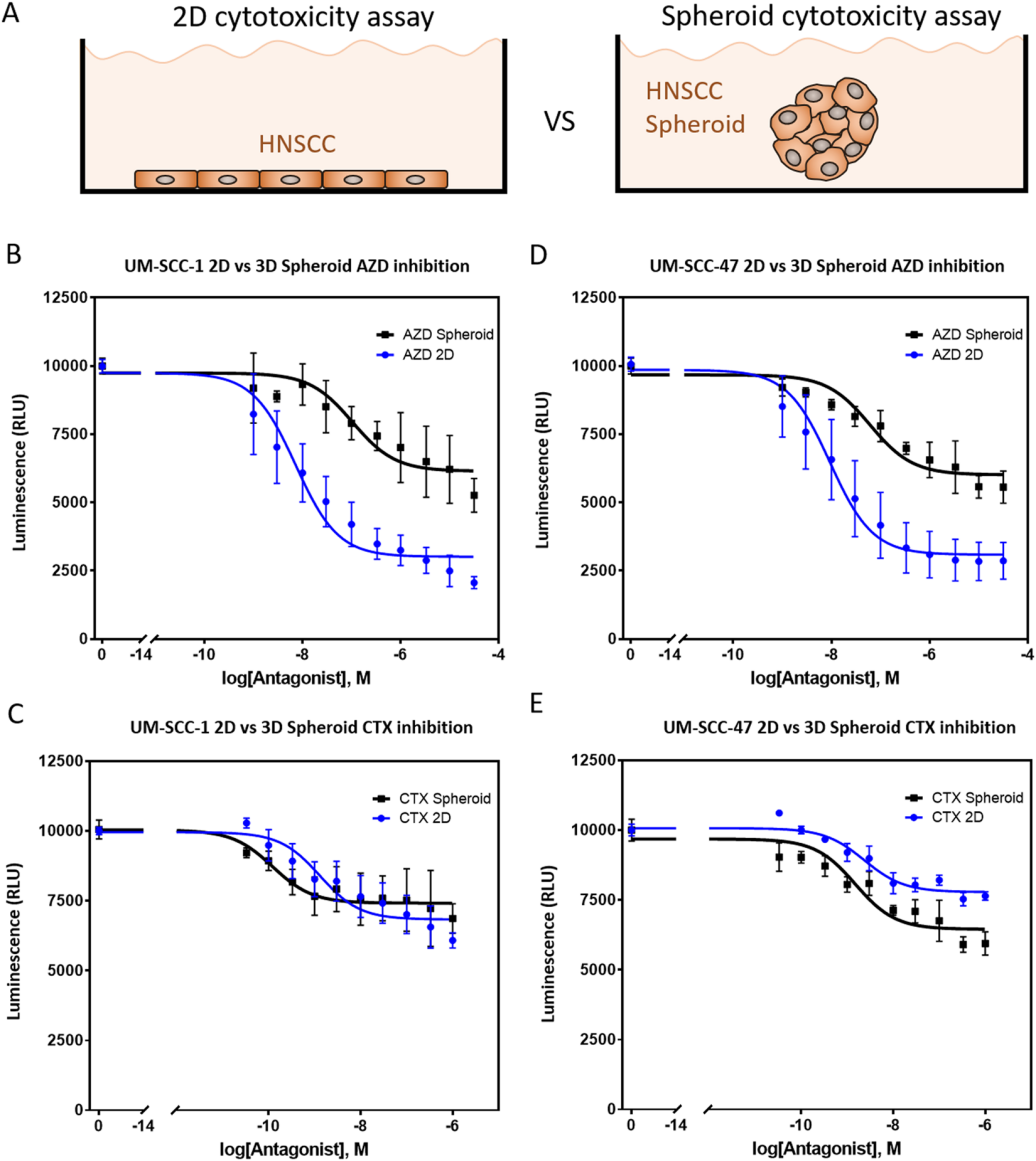
Spheroid drug response. (**A**) UM-SCC-1 or UM-SCC-47 were cultured in 2D on a plastic 96 well-plate or grown as a multicellular spheroid. The cell number and media volume were kept constant between the 2D and 3D experiments. (**B**) Graph shows UM-SCC-1 response to AZD8055 in 2D and in the spheroid. (**C**) Graph shows UM-SCC-47 response to AZD8055 in 2D and in the spheroid. (**D**) Graph shows UM-SCC-1 response to CTX in 2D and in the spheroid. (**E**) Graph shows UM-SCC-47 response to CTX in 2D and in the spheroid. Graphs show mean value ± standard deviation.

Finally, we studied the effect of culturing HNSCC cells as tumor spheroids in drug response. Spheroids provide a more complex model since they mimic the nutrient, hypoxia and waste product gradients observed *in vivo* in solid tumors (Fig. 3A). When grown as a spheroid, UM-SCC-1 cells exhibited a significantly higher resistance to AZD8055, increasing cell viability across the drug concentration range and reducing cytotoxicity 2.6-fold at highest doses, p-value < 0.001 (Fig. 3B). Interestingly, culturing UM-SCC-1 cells as a spheroid did not affect CTX sensitivity (p-value = 0.18); suggesting culture in a 3D hydrogel and a spheroid affect drug sensitivity differently (Fig. 3C). UM-SCC-47 cells cultured as a spheroid had significantly higher resistance to AZD8055; showing a 2-fold reduction in cytotoxicity at highest drug concentrations and significantly increased cell viability over the dose-response slope, p-value < 0.001 (Fig. 3D). Finally, when cultured as a spheroid UM-SCC-47 showed a slight but statistically significant increase in resistance to CTX treatment at higher drug concentrations, p-value = 0.01 (Fig. 3E). These results showed again the impact that the culture environment exerts in drug response.

**Figure 4:**
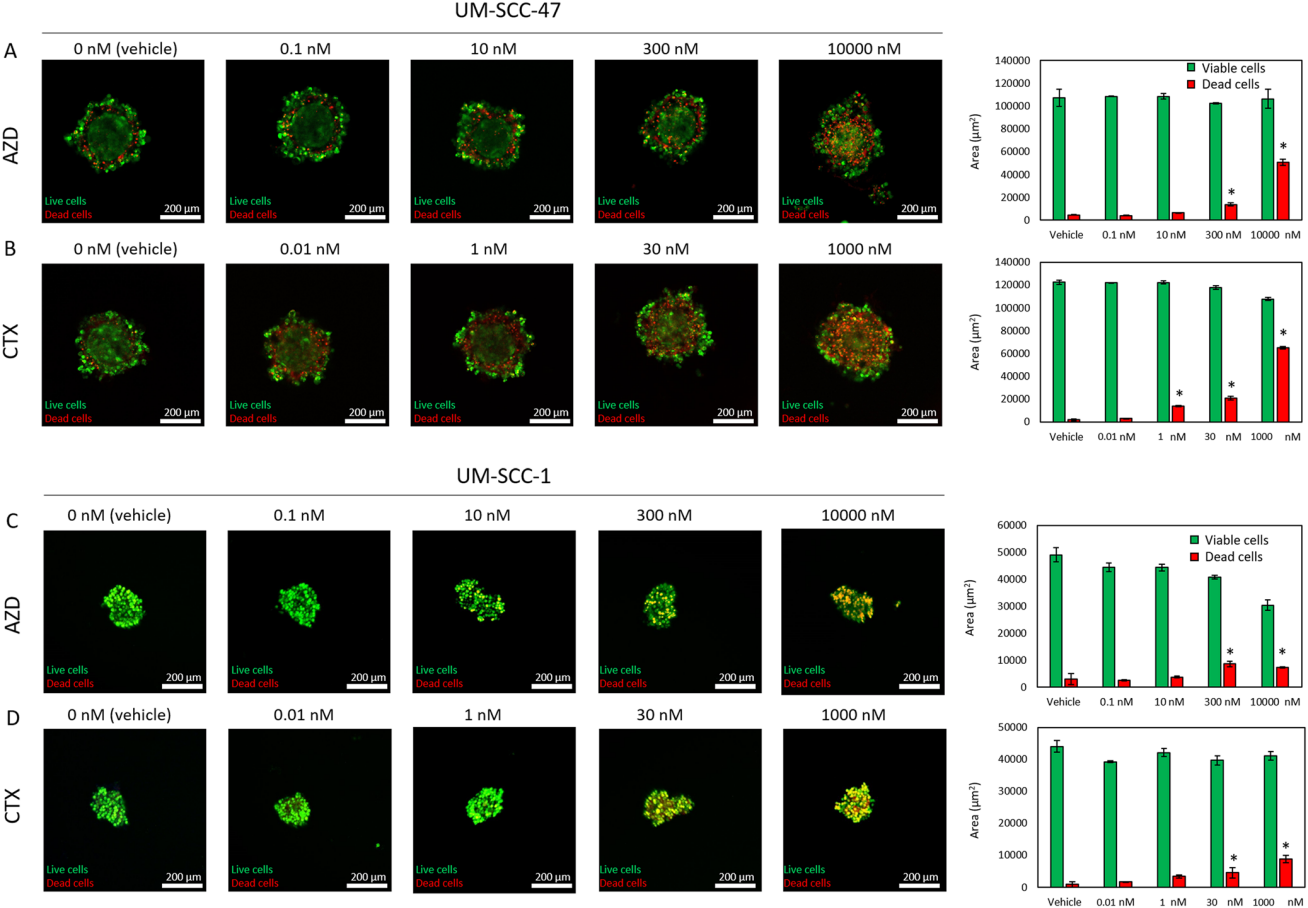
Spheroid differential response depending on cell location. AZD8055 and CTX response in spheroids was evaluated by confocal microscopy, labelling viable cells in green (CAM) and dead ones in red (propidium iodide). (**A**) Images show UM-SCC-47 cell response to AZD8055 at different concentrations. Graphs show the area occupied by viable and dead cells (green and red columns respectively). Graphs display mean ± standard deviation. (**B**) UM-SCC-47 response to CTX. (**C**) UM-SCC-1 response to AZD8055. (**D**) UM-SCC-1 response to CTX.

**Figure 5:**
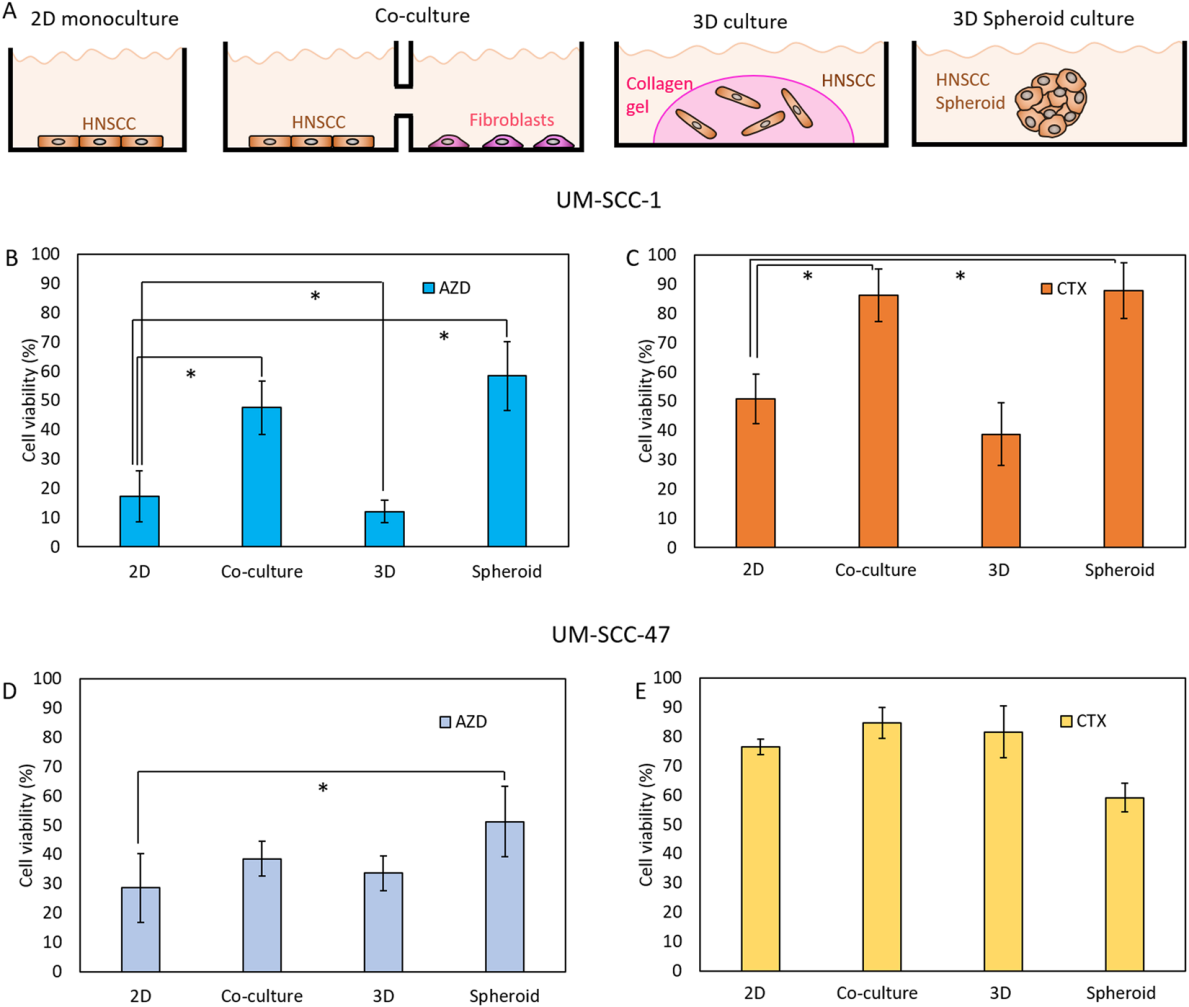
Influence of the culture environment in the drug response. The graphs show the cell viability in the different culture environments (**A**) in the presence of AZD8055; 10 uM for 72 hours (**B**) and CTX; 1 uM for 72 hours (**C**) for UM-SCC-1 and UM-SCC-47 cells **(D**,**E**). Graphs show mean value ± standard deviation. And asterisk denotes p-value < 0.05.

Tumor spheroids can exhibit a barrier effect, hindering drug penetration into the spheroid. Thus, we wanted to explore whether the increased drug resistance observed in spheroids was only due to a barrier effect. In order to study the penetration of small molecules (e.g., AZD), we used doxorubicin (Dox), a naturally fluorescent chemotherapy drug (Supplemental Fig. 1A–C). Additionally, we used a fluorescently labelled anti-EpCAM antibody with a similar weight to CTX to evaluate antibody penetration (Supplemental Fig. 1D–F). Although both compounds diffused into the spheroid, Dox exhibited a faster penetration into the spheroid. After 5 hours, Dox profile was flat, whereas the antibody profile exhibited a gradient across the spheroid diameter. Additionally, UM-SCC-1 and UM-SCC-47 spheroids were treated with AZD8055 and CTX and after 3 days cell viability was evaluated by confocal microscopy. The confocal images showed both AZD8055 and CTX killed UM-SCC-47 cells specially at the periphery of the spheroid, only affecting the core at the highest concentrations (Fig. 4A). On the other hand, AZD8055 and CTX showed a minor effect on UM-SCC-1 cells regardless their location in the spheroid (Fig. 4B). When combined, the results showed the culture environment can dramatically change HNSCC drug response (Fig. 5). Therefore, in order to develop new *in vitro* functional assay for HNSCC treatment, the variability generated by the culture environment is a challenge that these potential assays need to overcome.

## Conclusion and Discussion

HNSCC targeted therapy holds great potential and a large number of new potential therapies targeting EGFR are being explored^8^. However, most *in vitro* functional assays do not mimic the complexity observed *in vivo*^23,24^. Thus, to improve the predictive power of *in vitro* drug sensitivity assays and accelerate the translation of new therapies and assays to the clinic; the level of complexity included in these functional assays should be carefully considered^17^. In this work, the effect of the culture environment (i.e. monoculture vs co-culture, 2D vs 3D, 2D vs spheroids) has been assessed in HNSCC cells. The results showed the culture environment had a deep impact in drug sensitivity, leading to higher or lower sensitivity depending on the specific condition assessed. In this context, the presence of CAFs led to an increase in CTX and AZD8055 resistance in UM-SCC-1 cells; highlighting the role CAFs play in EGFR pathway^25^. Cell culture in a 3D hydrogel showed no change in drug response in UM-SCC-1. However, culturing the cells as a 3D spheroid led to a significant increase in drug resistance, showing a similar response compared with the addition of CAFs. Confocal images showed that UM-SCC-47 cells at the spheroid periphery were more affected by AZD and CTX compared with the cells at the core. Conversely, confocal images showed AZD and CTX exhibited no effect on UM-SCC-1 spheroid viability regardless their position within the spheroid. This observation suggested the increased drug resistance observed in UM-SCC-1 spheroids was not due to a barrier effect; but to mechanistic changes in the targeted pathways when cells were cultured as spheroids. Interestingly, the culture environment affected drug response on UM-SCC-1 (i.e. HPV-negative) as well as UM-SCC-47 (i.e. HPV-positive) cells, showing that response to anti-EGFR therapy is modulated by the environment in both HNSCC types. Therefore, HNSCC drug response is a complex process where multiple different factors influence drug response. Overall, this study demonstrated the deep impact that the culture environment has on HNSCC EGFR targeted therapy and highlights the challenge of heterogeneity in drug response. Multiple previous reports have analyzed the molecular mechanisms involved in drug sensitivity, including cetuximab and mTOR inhibitors^18,26,27^. Traditionally, most studies evaluating these molecular pathways have been performed in 2D monocultures. Recently, researchers have started to point out the effect the culture environment can exert in protein expression, leading to different drug response^28–30^. Thus, future studies could dissect these environment-dependent mechanistic alterations to identify more effective therapies against HNSCC. Additionally, future *in-vitro* work should pursue a model capable of predicting patient tumor response to drug therapy. This study utilizes components of the tumor microenvironment (i.e. extracellular matrix proteins, primary stroma cells, and cell-cell interaction) to better recapitulate *in-vivo* tumors; however, patient tumor cells are not always accurately represented by immortalized cell lines such as UM-SCC-1 and UM-SCC-47. For this reason, future studies should investigate models utilizing primary patient-derived tumor cells. In conclusion, the role of the tumor environment in HNSCC, and also in other types of cancer, should be considered if functional tests will be used in the clinic to predict drug response.

## Supplementary information

Supplementary information
