# Supplementary material for: Effects of culture method on response to EGFR therapy in head and neck squamous cell carcinoma cells
Source: Sci Rep. 2019 Aug 28;9:12480. doi: 10.1038/s41598-019-48764-3 (PMC6713778; doi:10.1038/s41598-019-48764-3)
Supplement: Supplementary file 1 — Supplementary information [file 41598_2019_48764_MOESM1_ESM.docx]

**Effects of culture method on response to EGFR therapy in head and neck squamous cell carcinoma cells**

Jose M. Ayuso^1,2,3*^, Ross Vitek^2,3*^, Adam D. Swick,^4^ Melissa C. Skala^1,2,3^, Kari B. Wisinski^3^, Randall J. Kimple^3,4^, Paul F. Lambert, David J. Beebe^2,3,5^.

1. Morgridge Institute for Research, 330 N Orchard street, Madison, WI, USA.
2. Department of Biomedical Engineering, University of Wisconsin, Madison, WI, USA.
3. The University of Wisconsin Carbone Cancer Center, University of Wisconsin, Madison, WI, USA.
4. Department of Human Oncology, University of Wisconsin, Madison, WI USA
5. Department of Pathology & Laboratory Medicine, University of Wisconsin, Madison, WI,USA

*These authors equally contributed to this work.

Corresponding author: David J. Beebe. E-mail: djbeebe@wisc.edu

Supplementary Figures


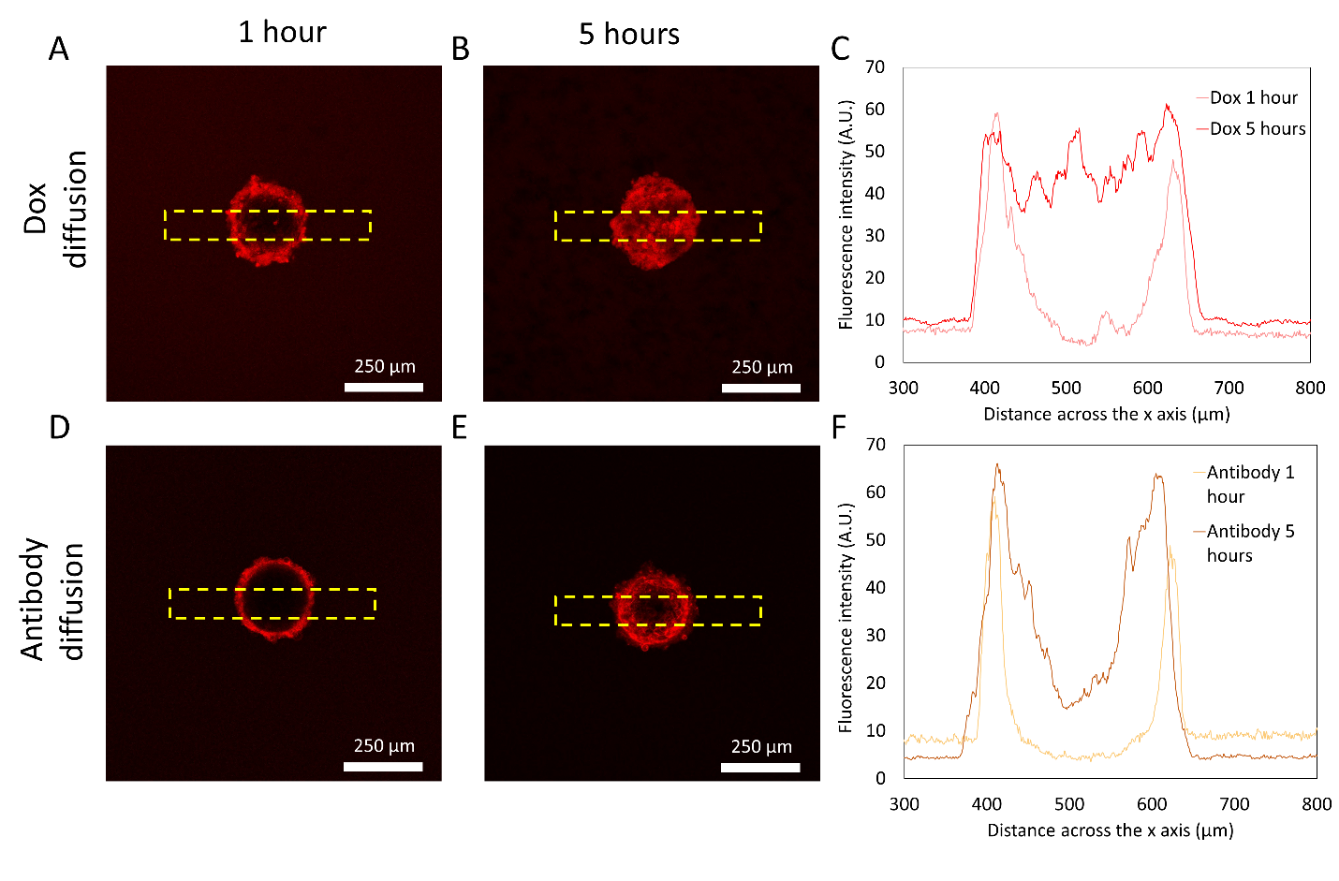


Supplementary Figure 1. Drug penetration. Small molecule (i.e., Doxorubicin) and antibody penetration (i.e., anti-EpCAM) in the UM-SCC-1 spheroids was analyzed by confocal microscopy in 1500 cells spheroids. A-B) Confocal images show the rapid penetration of the fluorescent drug doxorubicin after 1 and 5 hours. C) The graph shows the doxorubicin profile across the yellow rectangle in the confocal images. D-F) Similar experiment assessing the penetration of antibodies. A fluorescent anti-EpCAM was added to the media. Confocal images show antibody penetration.
